# Reigniting the institutional engine for pharmaceutical access: when policy experiments become new global evidence

**DOI:** 10.3389/fpubh.2026.1771961

**Published:** 2026-01-28

**Authors:** Jiajv Chen, Wei Li

**Affiliations:** 1 School of Law, Renmin University of China, Beijing, China; 2 School of Law, Jinan University, Guangzhou, China

**Keywords:** intellectual property, international coordination, payment systems, pharmaceutical access, policy experiments

## 1 The speed gap between science and institutions

Rapid advances in biomedical science are redefining the boundaries of “curability”. Novel methods like CRISPR-Cas and RNA technologies vastly expand the possibilities for targeted interventions in genomic functions, thus extending the concept of classical gene therapy toward novel genomic therapies (1). mRNA technology has transformed vaccine development cycles, while cell therapy has enabled long-term remission for some cancer patients. However, the global distribution of these scientific achievements remains highly uneven.

Only 43% of clinical trials conducted by 20 major multinational pharmaceutical companies take place in Low and Middle-Income Countries, with low-income countries accounting for less than 4% (2). R&D projects for priority diseases (such as malaria, tuberculosis, and leishmaniasis) dropped from 367 in 2022 to 253, a decrease of nearly one-third. Technological innovation has not automatically delivered universal benefits; instead, it has exposed the lag in institutional supply. This gap violates the core spirit of the WHO's Global Strategy and Plan of Action on Public Health, Innovation and Intellectual Property, which mandates that health gains should be equitably shared across all countries.

For decades, the institutional design governing pharmaceutical R&D, pricing, registration, and distribution has been tied to the logic of high-income markets. Patent exclusivity sustains high prices, healthcare payment systems struggle to cover the costs of one-time therapies, and the lack of mutual recognition in cross-border regulation artificially prolongs drug approval cycles. Science discovers medicines, but institutions determine whether they reach people. The future of pharmaceutical access hinges on institutions' ability to reconcile scientific innovation with social responsibility.

## 2 When innovation meets institutions: the triple paradox of pharmaceutical access

### 2.1 The publicness paradox of intellectual property

The patent system—defined under international IP law as a temporary monopoly to incentivize innovation—was originally designed to balance inventor rights and public welfare, yet it has evolved into a ‘knowledge barrier' in the biomedical field. IP rights can be too restrictive (limiting research), too broad (for strategic reasons), or too strong (difficult to license). Effective governance of IP is essential to maximize societal benefits, not just to generate new inventions (3). Currently, there are over 11,000 CRISPR-related patent families worldwide, covering more than 200 institutions, with intertwined ownership and complex licensing processes. Moreover, uncertainty over rights forces potential users to obtain licenses from multiple patent holders globally, creating the risk of “royalty stacking” (4). The Medicines Patent Pool (MPP) provides an institutionalized remedy for this issue. Since its establishment in 2010, the MPP has facilitated the generic production of the HIV drug Dolutegravir in over 100 countries, reducing its price from $1,000 per person per year to less than $100 and benefiting more than 20 million people (5). Knowledge sharing has proven that innovation incentives and public affordability are not opposites, but outcomes of balanced institutional design.

### 2.2 The payment paradox of high-priced therapies

The cost of scientific breakthroughs is rising rapidly. In 2024, the US Food and Drug Administration approved Lenmeldy, a gene therapy for metachromatic leukodystrophy, with a price tag of $4.25 million, making it one of the world's most expensive medicines (6). Although domestic CAR-T therapies in China are priced lower than their US counterparts, all marketed domestic cell therapy products cost over RMB 1 million per dose (7). The static payment model of healthcare systems struggles to cope with “one-time high-cost” therapies. The US Centers for Medicare & Medicaid Services launched the “Cell and Gene Therapy Access Model (CGT Access Model)” in 2025–2026, aiming to reshape payment logic through Outcome-Based Payment (OBP): pharmaceutical companies must partially refund payments if patients fail to achieve preset treatment outcomes (8). Several EU countries have adopted risk-sharing contracts and installment payment mechanisms (9). In China, national healthcare insurance negotiations and local pilot programs serve as the primary means to enhance therapy accessibility.

### 2.3 The collaboration paradox of evidence silos

OBP mechanisms rely on Real-World Evidence (RWE) to verify the efficacy and safety of medicines. However, despite the establishment of RWE collection systems in multiple countries, data mutual recognition remains a global shortcoming. The fragmentation of global RWE data limits cross-border collaboration and policy learning. Differences in data collection methods, quality control, and analytical standards across countries hinder effective cross-border efficacy comparisons and knowledge sharing (10). This “evidence silo” phenomenon restricts innovation in global pharmaceutical payment policies. While individual countries have promoted efficacy-linked payment models through RWE, the lack of unified international data standards makes it difficult to form cross-border consensus and sharing mechanisms. Consequently, data incomparability and insufficient mutual recognition have become key barriers to institutional innovation and global pharmaceutical access.

## 3 Global institutional experiments: when policies become evidence

Faced with the common challenges of pharmaceutical access, countries worldwide are exploring new governance pathways through institutional experiments. These practices are not isolated policy pilots but verifiable, transferable carriers of evidence in global health governance—their core value lies in using RWE as a link to transform institutional design into quantifiable, scalable public health solutions.

The EU's DARWIN EU network provides a model for “standardized evidence generation” in cross-border pharmaceutical governance. As a core platform led by the European Medicines Agency, the network not only integrates long-term follow-up data of over 180 million patients but also addresses the “evidence fragmentation” caused by regulatory differences among EU member states by establishing unified standards for data cleaning, efficacy assessment, and cost-effectiveness analysis.

The U.S. Sentinel Initiative highlights the value of “institutional flexibility supported by large-scale data.” Between 2000 and 2024, the platform has covered a total of 500.1 million unique patient identifiers. Its core breakthrough is upgrading RWE from a “safety monitoring tool” to a “regulatory pillar for OBP”, verifying the feasibility of OBP mechanisms in controlling the costs of high-priced therapies and providing an actionable technical pathway for global healthcare systems to address one-time high-cost treatments.

China's National Medical Products Administration RWE pilot in Boao Lecheng has broken the traditional barrier that “overseas medicines must complete local Phase III clinical trials to be marketed.” By collecting RWE from over 50,000 patients, the pilot provided the basis for the “conditional marketing authorization” of 23 overseas innovative medicines. This practice proves that, on the premise of ensuring RWE quality, late-developing regions can shorten drug access timelines through “data substituting for some clinical trials,” offering a “leapfrog development” reference model for regions with scarce clinical trial resources, such as Southeast Asia and Africa.

Japan's Ministry of Health, Labor and Welfare's “conditional approval + RWE re-evaluation” mechanism focuses on rare diseases. Given the small patient population and limited clinical data for rare diseases, this mechanism allows rare disease medicines to be approved based on preliminary data, with pricing and indication scopes dynamically adjusted through RWE. It not only ensures timely access to medicines for patients but also mitigates the risk of “ineffective treatment” through subsequent data, filling the evidence gap in the governance of niche diseases.

Ghana, a low-income country party to the TRIPS Agreement, has piloted a “MPP + local RWE monitoring” mechanism for antimalarial drugs. Leveraging the WTO's public health flexibilities and WHO prequalification standards, it links generic drug production to real-world efficacy data, cutting the time for affordable antimalarials to reach rural areas by 40% and setting a replicable model for West African nations (11).

While rooted in different political and economic systems, these institutional experiments collectively validate the core logic of “institutional verifiability”—when institutional design uses RWE as a feedback mechanism, it can transcend geographical adaptability constraints and become global evidence. Notably, these national practices align with critical frameworks from international organizations: they echo the WHO's call for cross-border health equity, leverage the WTO TRIPS Agreement's public health flexibilities, and reflect WIPO's guidelines on balancing patent protection with technology sharing. From the EU's “standardized consensus-building”, the U.S.'s “payment-regulation data linkage”, China and Japan's responses to “late-developing needs and niche areas,” to Ghana's adaptation for resource-scarce contexts, these practices cover diverse scenarios including high-income and low-to middle-income countries, common and rare diseases, and cross-border collaboration and regional innovation, forming a complementary evidence system. This pathway of “differentiated practice → common evidence → global adaptation” is the key to translating institutional innovation into public welfare.

## 4 Pathways to institutional synergy: making innovation more equitable

**Figure 1:**
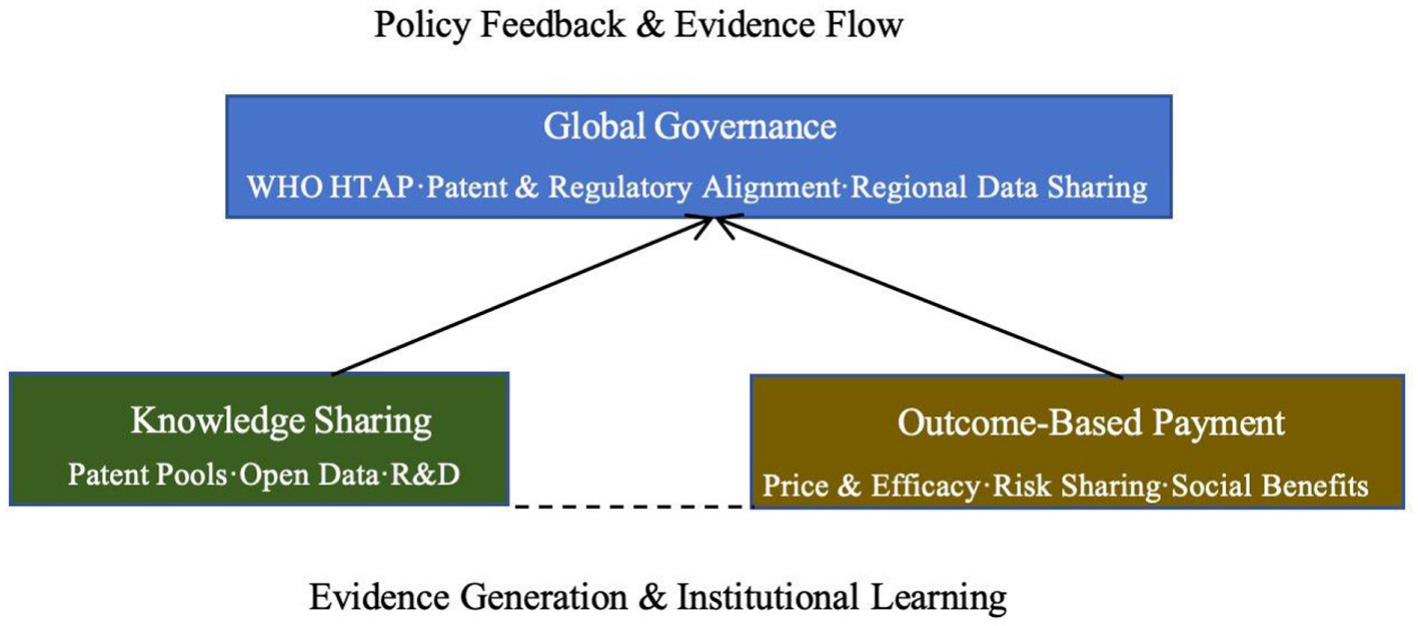
The sustainable institutional framework for pharmaceutical access model.

The Sustainable Institutional Framework for Pharmaceutical Access (SIFPA) is an evidence- and data-driven dynamic institutional learning model (See Figure 1). Through the interaction of data, incentive mechanisms, and governance structures, it enables institutions to continuously feed back, adjust, and optimize during implementation, thereby addressing global health challenges. The SIFPA emphasizes that the core of an institution lies not in one-time structural design, but in its ability to evolve continuously based on feedback mechanisms. Through systematic coordination and data-driven decision-making, the SIFPA accumulates evidence and forms a common language for cross-border collaboration, thereby advancing the equity and sustainability of global pharmaceutical access.

Critics argue that overemphasizing public health obligations may conflict with international commercial commitments (e.g., bilateral trade agreements prioritizing strict IP protection), but this framework resolves the tension by leveraging TRIPS flexibilities and outcome-based incentives that align corporate interests with global health goals. The framework is built on three pillars:

1. Knowledge Sharing Pillar
2. It promotes global innovation and technology transfer through patent pools, open licensing, and cross-sectoral R&D, lowering the entry barriers to innovation and enabling low-income countries to access therapeutic technologies more quickly.
3. OBP Pillar
4. It directly links pharmaceutical prices to their efficacy. Through multi-phase payment and risk-sharing mechanisms, it ensures that pharmaceutical prices are determined not only by R&D costs but also by the medicines' actual clinical value and social benefits.
5. Global Governance Pillar
6. It advances the mutual recognition of global pharmaceutical regulatory policies and data standardization, promotes coordination among national regulatory systems, and ensures the efficient flow and sharing of global resources.

Through the interaction of these three pillars, the SIFPA forms a self-reinforcing virtuous cycle. As pharmaceutical usage data and treatment outcomes are continuously collected, the basis for policy adjustments becomes more precise, helping decision-makers better understand the actual effects of different policy measures. The data-driven feedback mechanism allows policies to be adjusted in a timely manner, ensuring global pharmaceutical access. This framework also fosters cooperation and knowledge sharing among countries, promoting the coordination and unification of the global pharmaceutical governance system. By sharing experiences and data, countries can not only improve their own pharmaceutical access but also drive the achievement of global public health goals. Continuously optimized policy measures ensure equitable access to medicines and advance the sustainable development of global health.

## 5 Toward institutional innovation for global health equity

Achieving global pharmaceutical access is inseparable from the drive of institutional innovation. As global public health challenges intensify, traditional models of pharmaceutical management and distribution can no longer address increasingly complex needs, making institutional innovation a key factor in advancing global pharmaceutical access. The promotion of the SIFPA aims to drive the transformation of global governance models through policy synergy and cross-border cooperation. Through this framework, policymakers can collectively build a more equitable, transparent, and efficient pathway for global pharmaceutical access.

Under this framework, policymakers should focus on deepening and implementing the following aspects:

1. Strengthen Cross-Border Policy Coordination
2. Coordination and cooperation in global pharmaceutical access policies are crucial. Countries should, under the framework of the WHO and consistent with Sustainable Development Goal (SDG) 3 (Ensure healthy lives and promote well-being for all at all ages), promote resource sharing and policy experience exchange in the pharmaceutical supply chain. Meanwhile, aligned with the WTO's TRIPS Agreement flexibilities for public health, this coordination should address trade-related barriers to pharmaceutical access. A feasible plan is to establish a “Global Pharmaceutical Access Emergency Coordination Team” under the WHO, and develop a “cross-border allocation mechanism for vaccines/antiviral medicines” targeting public health emergencies such as COVID-19 and monkeypox. Led by G20 countries, this team will ensure a unified and robust global response mechanism during emergencies.
3. Promote the Linkage Between Pharmaceutical Prices and Efficacy
4. To achieve fairness and transparency in pharmaceutical pricing, the OBP model should become the core of global pharmaceutical pricing. By linking pharmaceutical prices to efficacy, market demand, and global health equity, countries can set reasonable prices based on the actual effects and cost-effectiveness of medicines in different populations. Examples include the U.S. CGT Access Model (pharmaceutical companies refund 50% if patients show no efficacy within 1 year) and EU risk-sharing contracts (CAR-T costs paid in five installments). This not only ensures universal access to high-efficacy medicines but also incentivizes pharmaceutical companies to develop more innovative medicines benefiting global health through a fair pricing mechanism.
5. Expand Knowledge Sharing and Technology Transfer
6. Realizing global health equity requires countries and enterprises to facilitate low-income countries' access to new medicines and therapeutic technologies through patent sharing, open licensing, and technology transfer—especially in the fields of anti-cancer, antiviral, and rare disease treatments. In this regard, efforts should be made to expand the coverage of the MPP from HIV medicines to anti-cancer and rare disease drugs, promote multinational pharmaceutical companies to establish local production bases in low- and middle-income countries, and transfer at least two core production technologies to local enterprises. By accelerating technology transfer and sharing innovative achievements, global innovative medicines and therapeutic technologies can benefit resource-scarce regions earlier, laying the foundation for global health equity.
7. Enhance Data Interoperability and Standardization
8. The efficient operation of pharmaceutical regulatory and healthcare payment systems relies on data support. Global pharmaceutical regulatory authorities and healthcare systems should promote cross-border data sharing and establish unified data standards, enabling countries to share actual data on pharmaceutical efficacy and cost assessment. A potential plan is for the WHO to lead the establishment of a “Global RWE Data Standards Alliance,” unifying data collection fields for pharmaceutical efficacy and safety (such as adverse reaction classification standards and efficacy evaluation time points) and promoting mutual recognition of data formats among member states. Additionally, a cross-border data sharing platform should be built to allow low- and middle-income countries to access non-confidential data free of charge, reducing their evidence acquisition costs for policy formulation. Ultimately, this will promote the optimization and coordination of global pharmaceutical policies.

Through the implementation of these institutional innovations, the SIFPA provides a new pathway for global health equity. The advancement of global pharmaceutical access should not rely solely on scientific breakthroughs but more on institutions' ability to adapt flexibly, cooperate coordinately, and continuously optimize. Only when the global governance system possesses the capacity for dynamic adjustment and self-optimization can pharmaceutical access become the norm in global health governance, ensuring that people in every country and region can equitably benefit from the achievements of global health progress.
